# PET/CT and the solitary pulmonary nodule

**DOI:** 10.1590/0100-3984.2020.53.2e2

**Published:** 2020

**Authors:** Erique Guedes Pinto, Diana Penha, Klaus L. Irion

**Affiliations:** 1 Consultant Radiologist, PhD Student, Universidade da Beira Interior, Covilhã, Portugal.; 2 Honorary Professor of Thoracic Imaging, The University of Manchester, Co-lead for Thoracic Imaging, Department of Radiology, Manchester University NHS Foundation Trust.

In the previous issue of **Radiologia Brasileira**, Mourato et al.^(1)^ reported the results of their research on the impact of ^18^F-fluorodeoxyglucose positron emission tomography/computed tomography (^18^FDG-PET/CT) on the characterization of pulmonary nodules as benign or malignant, comparing the Swensen model^(2)^ with the Herder model^(3)^. They found that, in their small cohort (n = 33), the Herder model (which includes ^18^FDG-PET/CT findings in the formula for predicting the probability of malignancy) changed the initial (Swensen model) classification of the probability of malignancy in over 50% of the cases. Although we recognize that the impact and generalizability of their observations are quite limited, the publication of their study inspired us to discuss the concept of diagnostic imaging replacing histopathological diagnosis.

The idea that a noninvasive imaging method could preclude the need for histopathological confirmation of the nature of a lesion in any human organ is quite tempting, driving researchers and funding agencies to pursue and fund research on the topic. The results of such research have a direct impact on patient care. In most such research, the diagnostic imaging method is tested against the histopathological diagnosis, the latter being accepted as the gold standard. Limiting our discussion to pulmonary nodules, various imaging modalities have been tested and studies have demonstrated that some methods, such as the assessment of the temporal contrast enhancement of nodules, ^18^FDG uptake on PET/CT, and diffusion or contrast enhancement curves on magnetic resonance imaging (MRI) scans, are highly accurate. Some of us remember a time when radiologists were expected to differentiate between adenocarcinoma or squamous cell carcinoma based on the characteristics of the lesions on chest X-rays. As odd as it may sound today, radiologists could fail the board examinations if they were unable to make this “diagnosis”. Obviously, science progressed, and this has proven to be a meaningless exercise, given that the modern imaging modalities are far superior to chest X-ray for this task. Radiology board examinations now test whether we can discern between benign or malignant nodules or even among different histopathological types of lung malignancy, based on their imaging characteristics on CT, PET/CT, or MRI. Of course, in many cases it is possible to suggest that a pure ground-glass nodule is more likely to be an adenocarcinoma than a squamous cell carcinoma and that an irregular endobronchial focal thickening is more likely to be a squamous cell carcinoma than an adenocarcinoma. However, the chance that these diagnoses could be wrong is not negligible and the impact that such misdiagnoses may have on individual patients raises questions regarding the appropriateness of attempts to make such differential diagnoses. It is likely that, in the near future, we will be criticizing ourselves, as the current understanding on the capabilities of these methods will also be proven futile, because genetic and molecular tests are going to be indispensable for individualized targeted therapies, which play an ever-increasing role. In addition, the impact of the diagnosis of a malignant lesion and the determination of its histopathological type under microscopy, even with immunohistochemical tests, may be questioned regarding its importance and the impact it may have on patient survival and well being. Non-invasive pulmonary adenocarcinomas are a good example of that. It is not uncommon to follow slow-growing ground-glass nodules for several years and to see the affected patients dying from causes other lung cancer. In many cases, there are multiple foci of probably malignant ground-glass nodules in several lobes in the same patient. How aggressive should doctors be in pursuing histopathological confirmation of the nature of these lesions? Should such patients be treated before signs of invasion or clinical symptoms emerge? Undoubtedly, a proportion of these patients will progress to clinically relevant lung cancer. However, at the moment, neither imaging findings nor the histopathological classification allow us to distinguish between those who will progress to clinically relevant malignancy and those who will live with an indolent cancer for years, eventually dying from other causes. Screening programs for lung, breast, colon, prostate, and thyroid cancer should take that into consideration. Can we confidently say that we are doing more good than harm by pursuing the early diagnosis of a malignancy that may not necessarily manifest during the lifetime of the patient? Does the evidence in the literature unequivocally support telling patients that undergoing the treatment we are offering them and ensuring the impact that such a diagnosis has on their health are actually in their best interests as individual patients?
